# Mitigating Algorithmic Bias in Cancer Site Classification Models

**DOI:** 10.1200/CCI-25-00250

**Published:** 2026-03-11

**Authors:** Abhishek Shivanna, Adam Spannaus, Jordan Tschida, John Gounley, Patrycja Krawczuk, Heidi Hanson

**Affiliations:** ^1^ Advanced Computing for Health Sciences, Oak Ridge National Laboratory, Oak Ridge, TN

## Abstract

**PURPOSE:**

Integrating artificial intelligence in cancer diagnostics has improved tumor classification beyond rule-based systems. Despite these advancements, these models may still encode demographic biases. We conducted a large-scale, applied bias-probing study of a deep learning–based cancer site classifier to quantify race information encoded in document embeddings. We then evaluated how performance changes when race-correlated embedding dimensions are removed in a post-training sensitivity analysis.

**METHODS:**

The cancer site classifier was trained using 3.5 million electronic cancer pathology reports from six of the National Cancer Institute's SEER registries. We trained a hierarchical self-attention network to generate 400-dimensional document embeddings. These embeddings were used to train two downstream, gradient-boosted decision tree classifiers: one to classify the cancer sites and another to predict racial categories. We identified overlapping features by intersecting the top 50 feature-importance rankings from the site and race models and computed their cumulative feature importance in each model. As a post hoc sensitivity analysis, we progressively pruned these overlapping dimensions, retrained the site model, and compared overall macro-F1 and accuracy, race-stratified macro-F1, and group fairness metrics on the basis of demographic parity and equalized odds before and after pruning.

**RESULTS:**

The analysis revealed minimal feature overlap between the cancer site and race prediction models, and the cumulative importance scores indicated a negligible influence of racial information on clinical predictions. Post-training pruning of overlapping features did not compromise the models' diagnostic accuracy, with a 0.07% loss in accuracy.

**CONCLUSION:**

Our findings demonstrate that HiSAN-generated embeddings from SEER data can be used effectively in cancer site classification without significant demographic bias influencing the outcomes. Post-training pruning therefore functions as a practical audit and sensitivity check.

## INTRODUCTION

Artificial intelligence (AI) has enormous potential for advancing precision medicine and public health surveillance. In oncology, AI models that use machine learning (ML) and deep learning techniques have shown proficiency in deciphering complex clinical data for accurate cancer case identification and classification.^1-4^ However, as more AI tools have been integrated into clinical decision making, concerns have emerged regarding their potential to inherit or amplify biases present in their training data.^5-10^

**CONTEXT**

**Key Objective**
To evaluate whether document embeddings for cancer site classification encode racial information and whether pruning race-correlated embedding dimensions alters classification performance or fairness.
**Knowledge Generated**
In SEER pathology reports, only a small set of embedding dimensions were jointly important for site and race prediction; pruning them had negligible impact on overall accuracy or race-stratified macro-F1, and fairness metrics changed very little, suggesting that race information is diffusely encoded and that simple post hoc pruning functions as a sensitivity check.
**Relevance *(U. Topaloglu)***
Integrating artificial intelligence (AI) into cancer diagnostics enables accurate tumor site classification without introducing significant demographic bias, as demonstrated by minimal overlap between race-related and clinical features. Post-training pruning of race-correlated dimensions maintains diagnostic performance, offering a practical approach for fairness auditing in clinical AI models.*
*Relevance section written by *JCO Clinical Cancer Informatics* Associate Editor Umit Topaloglu, PhD.

Historically, health care data and algorithms have often reflected biases present in their training data, thereby perpetuating demographic differences in health outcomes if not addressed.^11-14^ Such biases can originate from imbalanced data sets, algorithmic design choices, or the erroneous interpretation of sociodemographic variables as clinical predictors.^15-23^ Developing robust methods for identifying and mitigating these biases is essential to ensure that AI technologies benefit all populations.

Pronounced differences between races in incidence rates, prognosis, and treatment outcomes are seen in colon, lung, breast, ovarian, and prostate cancers. For example, prostate cancer mortality among Black/African American men is nearly twice that among White men.^24-28^ Biases in algorithms used for case ascertainment in clinical studies have the potential to magnify existing disparities. If these algorithms fail to adequately represent varying demographics, then they may perpetuate and exacerbate differences in clinical trial enrollment and cancer outcomes.^29^ Designing and using AI tools that ensure impartiality will lead to more accurate, ethical, and objective health care.^23,24,30-37^

This study used a population-based data set from six of the National Cancer Institute's SEER registries to train a hierarchical self-attention network (HiSAN)^1,38^ to classify reports by cancer site. The objectives were threefold: (1) evaluate the extent to which HiSAN-generated document embeddings for cancer site classification encode demographic information related to race, (2) identify features that are predictive for both site and race, and (3) assess whether targeted removal of these overlapping features alters site-classification performance. Our approach provides a large-scale, applied protocol for bias probing and post-training sensitivity analysis using a simple two-step process. By evaluating and pruning overlapping features, we probe whether site predictions rely on features that also predict race, with the overall goal to stress-test the model.

## METHODS

### Data Description

We used a population-based data set comprising 3,514,324 electronic pathology reports obtained from the Kentucky, Louisiana, New Jersey, Seattle, California and Utah cancer registries through the institutional review board protocol DOE000152. This data set included records from 1,083,352 unique patients collected systematically from the aforementioned SEER catchment areas. Each report contains a detailed record of tumor characteristics and the pathologists' notes. These reports were classified according to the International Classification of Diseases for Oncology, Third Edition (ICD-O-3), focusing on five classification tasks: (1) site, (2) subsite, (3) histology, (4) laterality, and (5) behavior.

### Data Preprocessing

**FIG 1.**
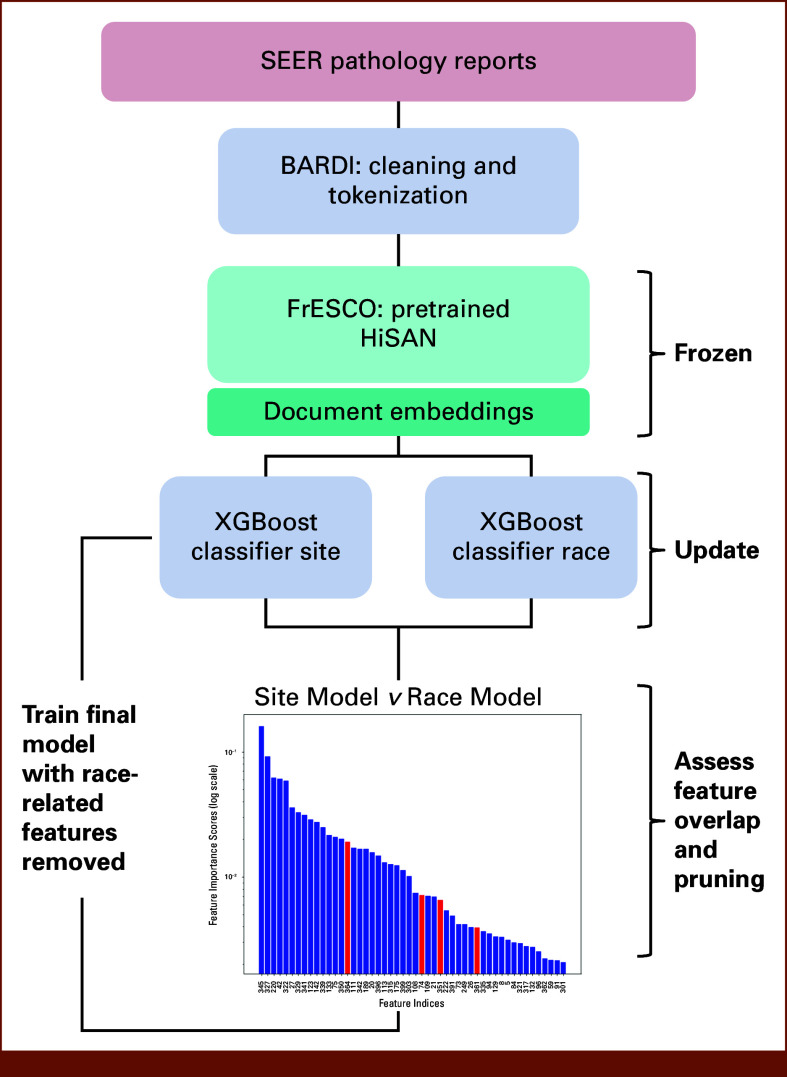
Flowchart of the data processing pipeline.

Clinical text was preprocessed using the Batch-processing Abstraction for Raw Data Integration (BARDI) framework,^38^ which standardizes report cleaning, tokenization, segmentation, and logging of all transformations. We used domain-specific regular expressions to normalize medical terminology and remove nonclinical content such as identifiers, URLs, and formatting artifacts, yielding deidentified, consistently formatted reports for downstream modeling. Although preprocessing can itself introduce subtle biases by changing how information is represented, a detailed audit of preprocessing effects is beyond the scope of this study. The data processing pipeline is summarized in Figure 1

### Generating Embedding Vectors Using a HiSAN

We used a multitask HiSAN model with self-attention to evaluate the text and generate 400-dimensional document embeddings for downstream classification. Detailed information about the HiSAN architecture is available elsewhere.^1,39^

### Initial Model Fit for HiSAN

The initial HiSAN fit demonstrated robust performance with micro-accuracies of 91.5% (site), 67.3% (subsite), 90.9% (laterality), 76.6% (histology), and 97.0% (behavior), indicating effective capture of patterns needed for these tasks. We then froze the HiSAN parameters before the eXtreme Gradient Boosting (XGBoost) training phase.

### Study Design

#### Model Training

We used the HiSAN-generated embeddings to train two downstream gradient-boosted decision tree (GBDT) models: (1) a 70-class primary site classifier and (2) a race classifier with categories White, Black, Pacific Islander, Native American, Asian, other, and unknown. This two-step approach allowed us to describe how race information might be captured within the embeddings and to probe potential biases in the HiSAN mode.

The GBDT models^40^ were trained in Python 3.11.3 using XGBoost 2.1.0, with a stratified 70/30 train-test split to preserve class proportions and multiclass log-loss (mlogloss) as the objective.

To further explore potential biases, we created binary classification data sets for five cancer types with known demographic disparities: breast, lung, colon, prostate, and ovarian. Inclusion and exclusion criteria based on ICD-10 and histologic codes are provided in the Data Supplement. For each cancer, we split the data set into cases with that cancer versus all other cancers and trained a separate XGBoost model using the same embeddings. Comparing feature-importance scores between these cancer-specific models and the race model allowed us to assess how embedding features relate to sociodemographic characteristics.

To move beyond feature overlap as the sole indicator of bias, we evaluated the downstream site classifier using both global fairness metrics and subgroup performance. For the 70-class site model, we computed macro-averaged F1 and accuracy and demographic-parity and equal-opportunity gaps across racial groups using a macro one-versus-rest formulation. We also calculated race-stratified macro precision, recall, F1, and accuracy before and after pruning overlap dimensions to assess whether pruning disproportionately degraded performance for any subgroup or materially affected common fairness criteria.

#### Feature Overlap

Feature importance in our models was calculated using XGBoost's built-in functionality,^40^ which assesses improvement in accuracy brought by a feature, as seen in Equation 1:

$$Importance\left(f\right)=\frac{\left(\underset{t\in T}{\sum }\Delta accuracy\left(f,t\right)\right)}{\left|T\right|}$$

where *f* is a feature, *T* is the set of trees, and Δ*accuracy*(*f, t*) is the accuracy improvement due to feature *f* in tree *t*.

We then rank the features from each model by importance and limit our analysis of feature overlap to the top 50 features from each.

We identified features that were important in both our site and race prediction models by intersecting their significant feature sets, as seen in Equation 2:

$$Overlap\left(M_{1},M_{2}\right)=\left|F_{1}\cap F_{2}\right|$$

where *M*_1_ and *M*_2_ are the models compared, and *F*_1_ and *F*_2_ are their respective sets of important features.

We interpreted overlapping features as embedding dimensions where race-related information might be captured within the representations and influence primary site classification. We summarized their aggregate impact within each model using cumulative feature importance (CFI; Equation 3), defined as the sum of the importance scores of overlapping features. Larger CFI values indicate greater overall influence of overlap dimensions on model predictions and therefore a potentially higher risk that race information affects site classification.

$$Cumulative\;Importance\left(M\right)=\underset{f\in F_{overlap}}{\sum }Importance\left(f,M\right)$$

Equation 3: where *F*_overlap_ is the set of overlapping features, and *M* is the model under consideration.

#### Post-Training Global Feature Pruning and Sensitivity Analysis

Features that are in the top 50 most important features for both predicting site and race were pruned to mitigate potential bias. The XGBoost models were retrained to evaluate the impact of pruning on predictive performance and fairness. We monitored accuracy and macro-F1, as well as demographic-parity and equal-opportunity (true-positive rate [TPR] and false-positive rate [FPR]) gaps across race groups, and inspected stratified performance by race to identify any subgroup that might be disproportionately affected.

### Bias Threshold Selection

We initially chose a 10% CFI threshold for the race model as a heuristic to flag potentially problematic overlap, treating overlap dimensions whose cumulative importance exceeded 10% of total race-model importance as a possible conduit for demographic information. To assess the robustness of this choice, we performed a threshold sweep from 5% to 15% of total race-model importance, progressively pruning overlap dimensions ranked by race importance and retraining the site model at each level. For each configuration, we recomputed macro-F1, accuracy, and fairness gaps (demographic parity and equal opportunity); full sweep results are provided in the Data Supplement.

## RESULTS

To assess the potential biases and effectiveness of the HiSAN-generated embeddings, we conducted a comprehensive analysis across site classification and race prediction tasks. This section presents our findings, beginning with a detailed overview of the data set and sample characteristics. We then provide an in-depth assessment of feature importance and the impact of post-training feature pruning on downstream model performance and fairness.

### Data Description and Sample Characteristics

Table 1 provides an overview of the data set used in this study and highlights the distribution of race. In terms of representation, the data set mostly contains records of White individuals (84.2%), followed by Black (10.3%) and Asian (3.5%) populations. Notably, the other, Native, Island, and unknown categories collectively represent <2% of the data set, a severe racial imbalance that both reflects the underlying SEER registries and limits our ability to estimate performance and fairness reliably for these small groups.

**TABLE 1.** Sample Description and Distribution of Race

Race/Ethnicity	No. of Cases	Percentage
White	2,957,901	84.2
Black	361,738	10.3
Asian	123,877	3.5
Island	11,108	0.3
Native	19,295	0.5
Other	7,932	0.2
Unknown	32,473	0.9
Total	3,514,324	100

The distribution of cancer site classes used to train the primary site classifier is provided in the Data Supplement (Tables S1 and S2).

### Shared Feature Assessment: Identifying Potential Demographic Bias

We identified 12 overlapping features that were among the top 50 most important features for both the 70-class site model and the race model. These overlap dimensions had a CFI of 0.4034 in the site model and 0.0649 in the race model, suggesting that although they are collectively important for site classification, they play a relatively small role in race prediction. To put this in context, the highest individual feature-importance score in the site classification model was 0.2312, meaning that the CFI of 0.4034 reflects the cumulative effect of multiple overlapping features rather than a single dominant dimension.

Using a 10% CFI threshold in the race model, none of the models exceeded this level, reinforcing the relatively low potential for bias via this specific overlap pathway (Table 2). In the site-specific models, overlapping features also demonstrated low influence on the race model. For instance, the breast cancer model's overlapping features had a CFI of 0.0302, with similar patterns for the lung, colon, ovarian, and prostate models (Table 2). Additional baseline model-performance metrics are provided in the Data Supplement (Tables S3-S8).

**TABLE 2.** Initial Assessment of Feature Overlap and Importance

Model 1	Model 2	No. of Overlapping Features	Cumulative Importance in Model 1	Cumulative Importance in Model 2
Site	Race	12	0.4034	0.0649
Breast	Race	7	0.8789	0.0302
Lung	Race	8	0.0239	0.0356
Colon	Race	10	0.0126	0.0468
Ovarian	Race	13	0.0734	0.0588
Prostate	Race	9	0.0235	0.0524

Table 2 shows the number of overlapping features between each cancer site classification model (model 1) and the race prediction model (model 2), along with CFI scores representing the total impact of overlapping features on predictions. Higher CFI indicates greater influence of these features on model performance. The table includes the overall cancer site classifier and site-specific models for breast, lung, colon, ovarian, and prostate cancers.

Consistent with the low overlap CFI, global fairness metrics for the baseline (unpruned) 70-class site model suggested that race-related disparities were not dominated by a small set of highly race-predictive embedding dimensions. Using macro one-versus-rest calculations, the average demographic-parity gap across race groups was approximately 0.019, and the average equal-opportunity TPR and FPR gaps were approximately 0.52 and 0.002, respectively. These values indicate substantial variation in recall across races for some cancer sites, but they arise in the context of very low feature overlap between the race and site models. Together with the race classifier's poor performance on minority classes (Data Supplement, Table S9), this suggests that race information is diffusely encoded across many embedding dimensions rather than concentrated in a small overlap set.

### Post-Training Feature Pruning as a Sensitivity Analysis

**FIG 2.**
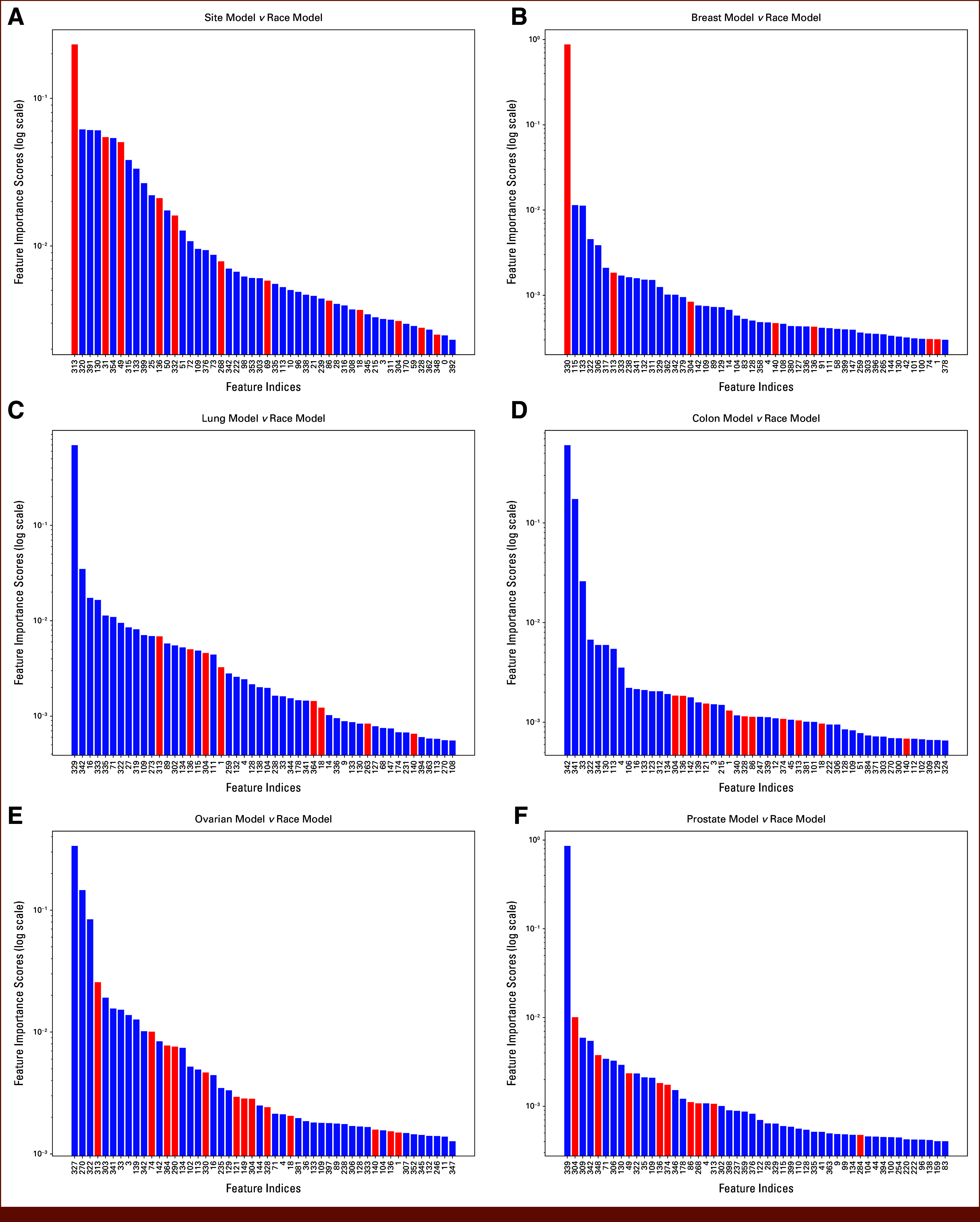
Feature importance scores from the XGBoost models for cancer sites on a logarithmic scale. The graphs represent (A) the overall site model, and the models for (B) breast, (C) lung, (D) colon, (E) ovarian, and (F) prostate cancers. Overlapping features that potentially indicate bias are highlighted in red. The *x*-axis represents feature indices, and the *y*-axis (log scale) quantifies the relative importance scores within each model, allowing for better visualization of features with lower importance scores.

**FIG 3.**
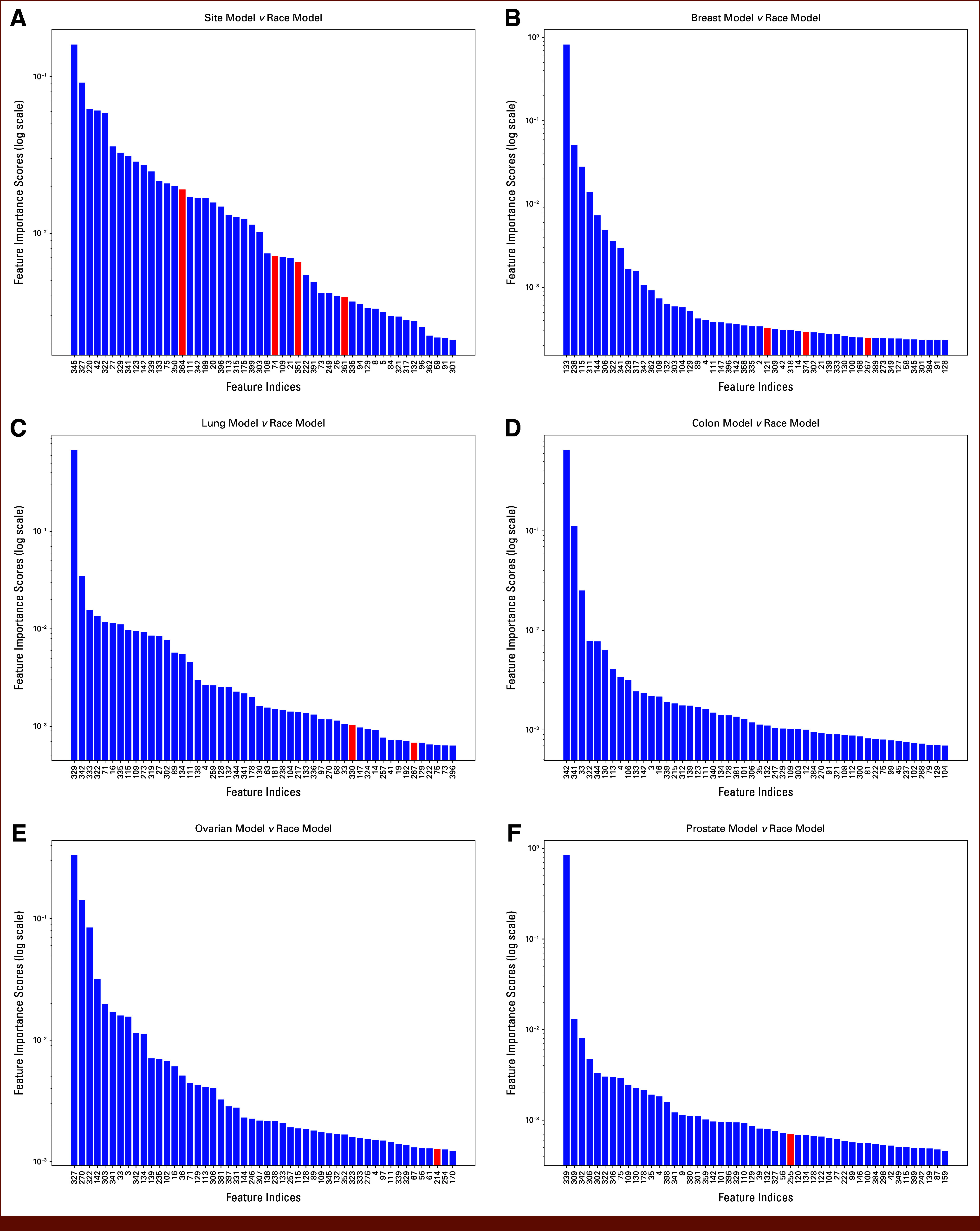
Feature importance scores across models after overlap pruning, plotted on a logarithmic scale. The graphs represent (A) the overall site model, and the models for (B) breast, (C) lung, (D) colon, (E) ovarian, and (F) prostate cancers. This figure mirrors the prepruning layout but shows the feature importance scores after mitigating bias by pruning the overlapping features. Sequentially, from breast to prostate cancer models and the cumulative site model, the absence of red bars signifies the elimination of shared influences, showing unbiased model performance. The *x*-axis lists the feature indices, and the *y*-axis (log scale) reflects their revised importance scores. The absence of red highlights indicates successful pruning of biased features to enhance the model's impartiality.

After targeted pruning of overlapping features, the number of overlap dimensions and their cumulative importance decreased across all models. Prepruning feature-importance patterns and the overlap dimensions identified for bias probing are shown in Figure 2. In the 70-class site versus race comparison, overlapping features dropped from 12 to 4, and their CFI in the site model fell to 0.0366. Similar reductions occurred in the site-specific models, indicating that the identified overlap dimensions are not essential to performance and can be removed without substantial loss in accuracy. We therefore view pruning as a sensitivity analysis that probes one plausible bias pathway rather than a complete mitigation strategy. For the colon model, pruning eliminated all overlapping features with the race model, whereas the ovarian and prostate models retained only one low-importance overlap dimension each (Fig 3, Table 3)

**TABLE 3.** Overlap and Importance Metrics After Feature Pruning

Model 1	Model 2	No. of Overlapping Features	Cumulative Importance in Model 1	Cumulative Importance in Model 2
Site	Race	4	0.0366	0.0655
Breast	Race	3	0.0009	0.0244
Lung	Race	2	0.0017	0.0165
Colon	Race	0	0.0000	0.0000
Ovarian	Race	1	0.0013	0.0035
Prostate	Race	1	0.0007	0.0072

Although none of the models met the 10% CFI threshold, we nevertheless pruned the overlapping features as an exploratory step to verify that diagnostic accuracy was not reliant on these dimensions. The largely unchanged performance after pruning suggests that site-specific models are primarily driven by clinically relevant information (Data Supplement, Tables S10-S17).

We next evaluated whether pruning overlap dimensions meaningfully changed fairness metrics or subgroup performance for the 70-class site model. Race-stratified macro-F1 results before and after pruning are provided in the Data Supplement (Table S18). Across a sweep of thresholds from 5% to 15% of the race model's total feature importance, macro-F1 and accuracy remained within 0.2 percentage points of the baseline, and demographic-parity and equal-opportunity gaps changed by at most 0.007 in absolute value (Data Supplement, Table S19). Notably, a configuration that removed approximately 5% of the race model's importance mass (9 dimensions) slightly improved both macro-F1 and fairness gaps, whereas removing all 12 overlap dimensions produced essentially identical performance and fairness to the baseline model.

To complement these global metrics, we computed race-stratified macro-F1 scores for site prediction before and after pruning all 12 overlap dimensions (Data Supplement, Table S18). For the three largest racial groups—White (n = 444,000), Black (n = 54,000), and Asian (n = 19,000)—the absolute change in macro-F1 was ≤1.1 percentage points. Smaller groups exhibited more variability: Native patients (n = 2,800) experienced an approximately 2.1-percentage-point increase in macro-F1, whereas the other category (n = 1,100) showed an approximately 6.5-percentage-point decrease. Given the small sample sizes for these groups, these estimates should be interpreted with caution. Overall, these analyses indicate that pruning the identified overlap dimensions does not substantially degrade performance for the major racial subgroups, but they also highlight that performance for underrepresented groups can shift by several percentage points even when global metrics and fairness gaps appear stable. In this sense, the pruning experiment should be interpreted as a stress test of one specific bias pathway rather than evidence that the model is fair across all racial groups.

## DISCUSSION

Pathology reports are intended to describe histologic characteristics of cancerous tissue and do not explicitly include demographic information such as race or ethnicity. However, because these reports are unstructured text, patient characteristics may still be indirectly reflected in the language used. We therefore examined whether features from our site prediction model inadvertently encode race-related information. We found minimal feature overlap between the cancer site and race prediction models, and overlapping features contributed much more to site than to race prediction (eg, CFI 0.4034 *v* 0.0649), suggesting that these dimensions are primarily clinically rather than demographically driven.

As a post-training sensitivity analysis, we pruned the identified overlapping features and reassessed both diagnostic accuracy and fairness. The largely unchanged performance and fairness metrics after pruning indicate that the HiSAN embeddings contain sufficient clinically relevant information for site prediction even when these overlap dimensions are removed. Race-stratified macro-F1 for the three largest racial groups (White, Black, and Asian) changed by at most 1.1 percentage points, with more variability in smaller groups (Data Supplement, Table S18). This pattern suggests that the pruning experiment is reassuring for the majority population but that our ability to precisely quantify its impact for underrepresented groups is limited by small sample sizes. However, these findings do not eliminate the risk of perpetuating biases, because race information appears to be distributed across many embedding dimensions and because fairness can be affected by factors beyond the specific overlap set we studied.

The limited change in demographic-parity and equal-opportunity gaps across a wide range of pruning thresholds (Data Supplement, Table S19) further suggests that, in this data set, race information is not concentrated in a small number of high-importance dimensions. Instead, race-related signals are likely encoded in a diffuse manner across the embedding space. This interpretation is consistent with the performance of the race model itself: despite an overall accuracy of 84%, the model performs poorly for minority classes, with the White population being the predominant and best-predicted class (Data Supplement, Table S9). The feature-importance scores for the race model are widely distributed, indicating that no single feature directly captures race information, in contrast to scenarios in which explicit mentions of sociodemographic variables result in dominant feature vectors.

A central limitation of this study is the severe racial imbalance of the SEER pathology data set: 84.2% of reports are from White patients, whereas Native, Island, other, and unknown categories each constitute well under 1% of the sample. Because the race model is trained predominantly on White patients, it has relatively few examples from which to learn stable patterns for minority groups, which is reflected in its very low recall and F1-scores for these classes (Data Supplement, Table S9). Fairness metrics such as demographic-parity and equal-opportunity gaps rely on accurate estimates of group-specific prediction rates; when the underlying group counts are small, these estimates become noisy and small changes in predictions can appear as large swings in the gaps. Our stratified site-model results (Data Supplement, Table S18) show that performance changes for small groups can be several percentage points even when global metrics remain flat, and we therefore caution against overinterpreting fairness for these subgroups. In future work, balanced or reweighted training schemes, or targeted oversampling of underrepresented groups, could provide a more robust setting for fairness evaluation, although such designs would trade off representativeness of underlying population prevalences.

Another limitation is that the overlapping features we prune are abstract embedding dimensions rather than interpretable clinical variables. Although we can quantify their impact on model performance and fairness, we cannot directly map them to human-readable concepts such as specific phrases or histologic patterns. As a result, we cannot say whether pruning removes clinically meaningful but demographically correlated signals, or merely statistical noise. Future work could pair this framework with concept-based interpretability methods or token-level attribution to better understand what information these dimensions encode.

Our methodology provides a practical framework for evaluating and stress-testing one pathway for demographic bias in AI models and is applicable to other demographic and socioeconomic variables beyond race. By training separate outcome and demographic classifiers on shared embeddings, identifying overlap dimensions, and testing the impact of pruning those dimensions, we can probe whether models rely on representations that are simultaneously predictive of clinical targets and demographic attributes. However, achieving impartiality in AI-driven diagnostics will ultimately require incorporating fairness considerations and constraints during the training phase itself, so that clinically relevant correlations are preserved without relying on protected characteristics.

Our investigation of HiSAN-generated embeddings for cancer site classification provides a two-step framework for evaluating and stress-testing demographic bias in phenotype classification algorithms. Within the constraints of a heavily imbalanced SEER data set, feature overlap related to race appears minimal and does not substantially influence site-classification performance or the fairness metrics we examined. These findings underscore the need for model-agnostic approaches to validate results across different ML platforms and data sets. Exploring advanced neural pruning and representation-learning techniques that diminish demographic signals in shared embeddings may further enhance the precision and robustness of AI diagnostics.

By focusing on a given variable such as race, we provide a context-specific rather than absolute explanation. Training separate models on shared embeddings and performing sensitivity tests to identify features important to both allow us to infer correlations related to race; if certain cancer types are highly correlated with a particular race, the model will likely reflect this, albeit inadvertently. This highlights the need for models that distinguish between clinically relevant information and demographic correlations without introducing bias.

Taken together, this study provides a practical method for evaluating demographic bias and testing simple post hoc pruning strategies in AI models used in cancer diagnostics, while highlighting that more sophisticated, training-time interventions will be required to fully address residual biases and to promote more consistent and equitable AI applications in health care.
